# Alteration of Endothelin 1, MCP-1 and Chromogranin A in patients with atrial fibrillation undergoing pulmonary vein isolation

**DOI:** 10.1371/journal.pone.0184337

**Published:** 2017-09-08

**Authors:** K. Lackermair, S. Clauss, T. Voigt, I. Klier, C. Summo, B. Hildebrand, T. Nickel, H. L. Estner, S. Kääb, R. Wakili, U. Wilbert-Lampen

**Affiliations:** 1 Department of Medicine I, Klinikum Grosshadern, University of Munich (LMU), Munich, Germany; 2 DZHK (German Centre for Cardiovascular Research), Partner site Munich, Munich Heart Alliance, Munich, Germany; 3 Department of Cardiology and Vascular Medicine, West-German Heart and Vascular Center Essen, University of Essen Medical School, University Duisburg-Essen, Essen, Germany; University of Minnesota, UNITED STATES

## Abstract

**Background:**

The relation between arrhythmias and stress is known. The aim of our current study was to elucidate whether plasma levels of previously described stress parameters are altered in highly symptomatic patients with atrial fibrillation (AF) per se and in patients undergoing ablation therapy by pulmonary vein isolation (PVI).

**Methods:**

96 patients with AF undergoing PVI were recruited. Plasma levels of Endothelin-1 (ET-1), MCP-1 and Chromogranin-A (CGA) were measured before and three months after ablation completed with clinical follow-up with respect to AF recurrence. Additionally, we examined 40 healthy age- and sex-matched volunteers as a reference.

**Results:**

Symptomatic AF patients showed increased levels of ET-1 compared to healthy controls (2.62pg/ml vs. 1.57pg/ml; p<0.01). Baseline levels of ET-1 were higher in patients presenting with AF after PVI (2.96pg/ml vs. 2.57pg/ml;p = 0.02). The temporal comparison revealed decreased ET-1 levels in patients without (2.57pg/ml vs. 2.33pg/ml; p<0.01) and unchanged ET-1 levels in patients with AF after PVI. Baseline MCP-1 was increased in AF patients vs. controls (268pg/ml vs. 227 pg/ml; p = 0.03). Both groups, with and without AF after PVI, showed an increase of MCP-1 compared to baseline (268pg/ml vs. 349pg/ml;p<0.01; 281pg/ml vs. 355pg/ml;p = 0.03). CGA was lower in AF patients compared to healthy controls (13.8ng/ml vs. 25.6ng/ml;p<0.01). Over time patients without AF after PVI showed an increase of CGA (14.2ng/ml vs. 20.7ng/ml;p<0.01). No change was observed in patients with AF after PVI.

**Conclusion:**

Our study demonstrated dysregulated levels of ET-1, MCP-1 and CGA in symptomatic AF patients. We could demonstrate an association between ET-1 to presence or absence of AF. Furthermore, we could show that a decrease of ET-1 as well as an increase of CGA after PVI, representing a trend towards control cohort levels, were both associated with restoration of sinus rhythm. These results provide new insights into the role of stress-related biomarkers in AF and AF treatment by ablation therapy.

## Introduction

Atrial fibrillation (AF) is the most common arrhythmia in clinical practice affecting more than six million Europeans [1,2]. AF is associated with increased morbidity and mortality since it is a major risk factor for thromboembolism [2]. Treatment of AF includes pharmacological (antiarrhythmic drugs) and interventional (pulmonary vein isolation, PVI) strategies but still remains challenging. The pathophysiological background of AF is complex and still incompletely understood [3,4]. Recently, it has been shown that physical or emotional stress can provoke AF and vice versa [5–10]. Stress cannot be measured directly, but several substances in the blood can serve as surrogate parameters for stress [11]. In this study we sought to investigate a potential relationship and pathophysiological role of three stress surrogate markers (Endothelin-1, monocyte chemotactic protein-1 and chromogranin A) in AF.

Endothelin-1 (ET-1) is produced by endothelial cells, smooth muscle cells, monocytes and macrophages after stimulation by catecholamines, cortisol, hypoxia, or interleukins [12]. Increased levels of Endothelin-1 were described in patients with different cardiovascular diseases [12]. Furthermore, Endothelin-1 has been reported as a biomarker for stress associated acute cardiovascular diseases. Recently, we could demonstrate that patients having an acute cardiovascular event in context of emotional stress showed significantly higher serum levels of Endothelin-1 compared to patients with acute cardiovascular events not having experienced a kind of emotional stress [10,11].

Similar findings have been described for monocyte chemotactic protein-1 (MCP-1) [10,11]. MCP-1 is a chemokine synthesized mainly in monocytes and macrophages Several factors affect synthesis of MCP-1 e.g. TNFα, Interferon-γ, PDGF and Angiotensin that are inducing synthesis of MCP-1 via NF-kB [13], which are also known to play a role in AF pathophysiology [4].

Chromogranin A (CGA) is an essential part of secretory vesicle in endocrine cells, neurons and neuroendocrine cells. As a part of secretory vesicles CGA is released with the content of the vesicle [14,15]. The majority of circulating CGA in healthy people is derived from adrenal medulla. There is also evidence that increased levels of CGA are associated with stress as we could previously show high levels of CGA after physical stress after extreme exercise [16].

The aim of the present study was to evaluate a potential association of the circulating Endothelin-1, MCP-1, and CGA with AF in highly symptomatic patients as well as to investigate changes of these markers in AF patients undergoing ablation therapy with respect to AF recurrence.

To test this, we measured the plasma levels of Endothelin-1, MCP-1, and CGA in AF patients undergoing PVI before and three months after ablation and compared these to healthy controls.

## Methods

### Patients and clinical follow up

96 consecutive patients undergoing PVI in the University Hospital Grosshadern were analysed in our study. To compare baseline levels, 40 age and sex matched patients without any history of atrial fibrillation or cardiovascular disease were selected as “healthy volunteers”. The analysed specimens were collected in a general biobanking effort that was continuously approved by the local ethics committee of the University of Munich and the Ethics Commission of the State Chamber of Medicine in Bavaria, Germany over the last years (reference numbers of the ethics approvals: most recent 494–16; 240–12; 04154; 166/01).

Clinical evaluation of every patient was performed before and 3 months after PVI including medical history, EHRA score, physical examination, blood collection, echocardiography, ECG, and Holter ECG. AF freedom was assessed by 24 hour/7 day Holter ECG or clinical AF-related symptoms (e.g. palpitations, tachyarrhythmia) three month after PVI. AF recurrence was defined as any atrial tachyarrhythmia lasting >30 seconds documented by ECG [17].

### Blood collection

Blood was collected via a direct venous puncture into 9 ml EDTA tubes (Sarstedt Monovette) and was processed for plasma isolation within 4 hours of collection. Blood was processed by spinning at 4000 rpm for 20 minutes at room temperature. Plasma was carefully transferred to a fresh RNAse/DNAse free tube and stored at -80°C.

### PVI procedure

Ablation was performed using a standardized clinical protocol (circumferential isolation of all pulmonary veins without additional ablation lines or CFAE ablation) as previously described [18]. In brief, all patients received sedation with a benzodiazepine and local anaesthesia. Via Seldinger technique three 8F sheaths were inserted into the femoral vein (coronary sinus (CS) catheter, ablation catheter and lasso catheter). After placement of all catheters and transseptal puncture anatomic visualisation was performed by angiography and CARTO 3D Mapping. PV potentials were measured by inserting the lasso catheter in each pulmonary vein. Then electrical PVI was performed using circumferential radiofrequency energy point-by-point ablation at the ostial site of each PV until achieving the procedural endpoint of electrical isolation of the PVs documented by entrance block. Electrical entrance block between the PV and left atrium and exit block if applicable was re-evaluated after a 30min waiting period confirming the procedural success.

### Biochemical assay

The plasma concentrations of Endothelin-1, MCP-1, and CGA were measured by a duoset enzyme linked immunosorbent assay (ELISA) (ET 1 and MCP: R&D System GmbH, Wiesbaden, Germany; CGA: IBL International, Hamburg, Germany).

### Statistical analysis

Statistical analysis was performed with SPSS 21. For not normally distributed variables we performed Wilcoxon Test for paired variables and Mann-Whitney-U test for non-paired variables. For dichotomous variables we used Fisher-Yates test. All values mentioned in the results section are medians unless otherwise listed. In addition, linear regression analysis adjusting for age, sex, diabetes, creatinine, hypertension and coronary artery disease was performed for Entothelin-1 baseline levels. Results are presented using box-plots showing median as well as interquartile ranges. A p value lower than 0.05 was considered statistically significant.

### Ethics statement

The study was performed on blood samples that were collected in a general biobanking effort that was continuously approved by the hospital’s ethics committee of the University of Munich (LMU) and the Ethics Commission of the State Chamber of Medicine in Bavaria (BLAEK), Germany over the last years (reference numbers of the ethics approvals: most recent 494–16; 240–12; 04154; 166/01). All procedures were performed according to the 1975 Declaration of Helsinki. Every participant provided written informed consent before participation, all samples were anonymized and processed according to the guidelines the local ethics committee of the University of Munich (LMU), Germany.

## Results

### Patient characteristics

In total, we included 96 AF patients in our study (Table 1). Within the AF population mean age was 61.8±10.9 years and 60.4% of the patients were male. 71.9% of the patients had paroxysmal AF, 28.1% had persistent AF. 81.2% of the patients treated by ablation showed no signs for AF recurrence three months after PVI. In addition, 40 age- and sex-matched healthy volunteers were included for baseline comparison of blood levels.

**Table 1:** Patient characteristics.

	AF study population	Healthy volunteers	p value
**Number of patients**	96	40	
**Demographics**			
age (years)	61.8±10.9	60.2±12.58	0.52
male sex (n, %)	58 (60.4%)	21 (52.5%)	0.45
**Type of AF**			
paroxysmal AF (n, %)	69 (71.9%)		
persistent AF (n, %)	27 (28.1%)		
**Cardiovascular Risk Factors**			
Hypertension (n, %)	64 (66.7%)		
Diabetes mellitus (n, %)	6 (6.3%)		
Hypercholesterolemia (n, %)	15 (15.6%)		
**Concomittant diseases**			
Coronary Artery Disease (n, %)	19 (19.8%)		
Renal impairment (GFR<60ml/min; n, %)	24 (25%)		
**Previous antiarrhythmic treatment**			
Ablation	10 (10.4%)		
Drug Treatment	96 (100%)		
Class I antiarrhythmic drugs	36 (37.5%)		
Class III antiarrhythmic drugs	20 (20.8%)		
**Echocardiographic parameters**			
LA diameter (PLAX, mm)	42±6.5		
Ejection fraction (%)	61.5±7.7		

For a further analysis we separated our AF patient cohort into two groups: 1) patients with recurrence of AF and 2) patients without recurrence of AF three months after ablation (Table 2). Both groups did not differ significantly regarding age (p = 0.95), sex (p = 1.00), type of AF (p = 0.95), previous ablation (p = 0.92), CAD (p = 0.7), or pre-treatment with antiarrhythmic drugs (p = 1.00).

**Table 2:** Clinical characteristics of patients with and without AF recurrence.

	AF recurrence	No AF recurrence	p-value
**Number of patients**	18 (18.8%)	78 (81.2%)	
**Demographics**			
age (years)	62.6±10.3	61.8±11.1	p = 0.95
male sex (n, %)	11 (61.1%)	47 (60.3%)	p = 1.00
**Type of AF**			
paroxysmal AF (n, %)	13 (72.2%)	57 (73.1%)	p = 0.95
persistent AF (n, %)	5 (26.8%)	21 (26.9%)	p = 0.95
**Cardiovascular Risk Factors**			
Hypertension (n, %)	14 (77.8%)	50 (64.1%)	p = 0.22
Diabetes mellitus (n, %)	1 (5.6%)	5 (6.4%)	p = 0.89
Hypercholesterolemia (n, %)	3 (16.7%)	12 (15.4%)	p = 0.89
**Concomitant diseases**			
Coronary Artery Disease (n, %)	3 (16.7%)	16 (20.5%)	p = 0.7
Renal impairment (GFR<60ml/min; n, %)	6 (33.3%)	18 (23.1%)	p = 0.4
**Previous antiarrhythmic treatment**			
Ablation	2 (11.1%)	8 (10.2%)	p = 0.92
Drug Treatment	18 (100%)	78 (100%)	p = 1.00
Class I antiarrhythmic drugs	5 (27.8%)	31 (39.7%)	p = 0.32
Class III antiarrhythmic drugs	4 (22.2%)	16 (20.5%)	p = 0.87
**Echocardiographic parameters**			
LA diameter (PLAX, mm)	42±7	43±5	p = 0.34
Ejection fraction (%)	58±12.7	62±6	p = 0.37

### Endothelin-1 (ET-1) plasma levels

**Fig 1:**
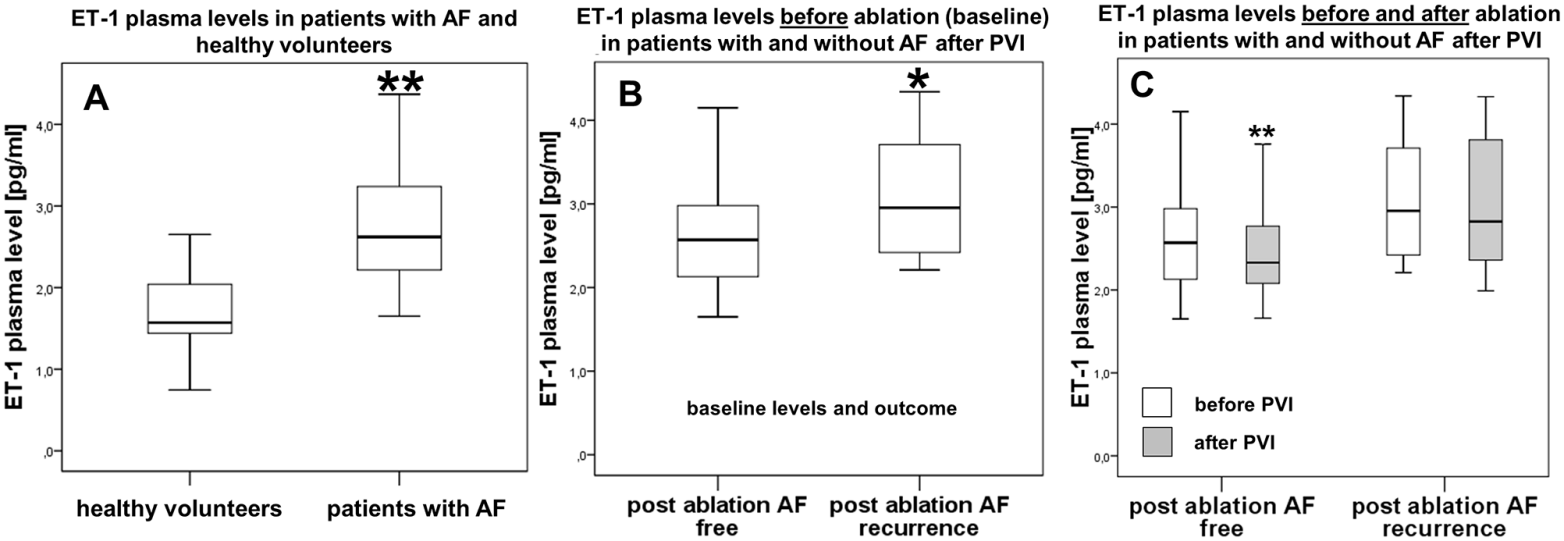
Plasma levels of Endothelin-1 [pg/ml]. **A**, Healthy reference group without AF (left) vs. all AF study patients (right). **B**, Baseline Endothelin-1 levels in patients without (left) and with AF recurrence (right) three months after ablation. **C**, Endothelin-1 plasma levels before and after PVI in patients without (left) and patients with AF recurrence (right). Baseline levels presented in white bars, levels three months after ablation presented in grey bars. * p<0.05, ** p<0.01.

To evaluate Endothelin-1 (ET-1) as a potential biomarker for AF we measured ET-1 plasma levels at baseline in patients with AF in comparison to healthy volunteers without AF. Patients with AF showed significantly higher levels of ET-1 compared to age- and sex-matched healthy volunteers without history of AF (2.62 pg/ml vs. 1.57 pg/ml; p<0.001; Fig 1-A).

Further analysis within the AF ablation cohort with respect to AF recurrence revealed that a significantly lower level of ET-1 prior ablation was associated with freedom of AF in the follow up period of 3 month (2.57 pg/ml vs. 2.96 pg/ml; p = 0.02; Fig 1-B). A regression analysis adjusting for age, sex, diabetes, creatinine, hypertension and coronary artery disease could confirm this statistically significant difference (p = 0.029). Patients without AF recurrence demonstrated a further decrease of ET-1 levels three months after ablation getting closer to the level of the healthy volunteers (2.33 pg/ml vs. 2.57 pg/ml; p<0.01) whereas ET-1 levels in patients with AF recurrence remained unchanged at an elevated level (2.83 pg/ml vs. 2.96 pg/ml; p = 0.09; Fig 1-C).

### MCP-1 plasma levels

**Fig 2:**
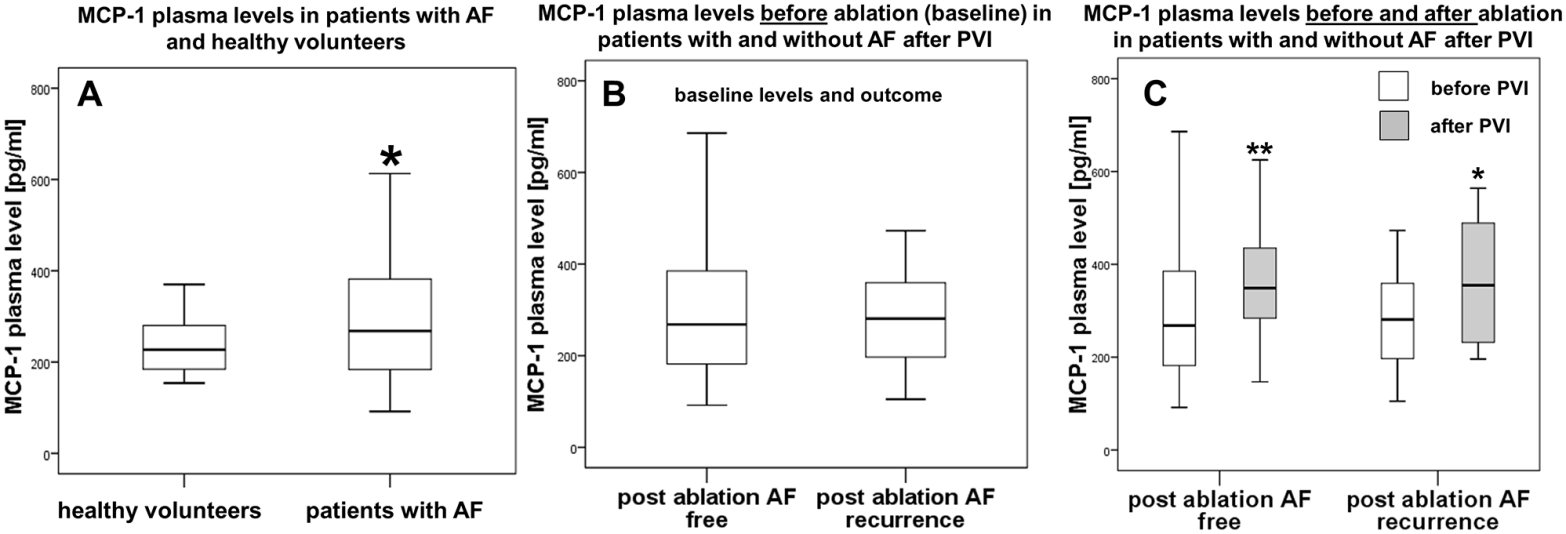
Plasma levels of MCP-1 [pg/ml]. **A**, Healthy reference group without AF (left) vs. all AF study patients (right). **B**, Baseline MCP-1 levels in patients without (left) and with AF recurrence (right) three months after ablation. **C**, MCP-1 plasma levels before and after PVI in patients without (left) and patients with AF recurrence (right). Baseline levels presented in white bars, levels three months after ablation presented in grey bars. * p<0.05, ** p<0.01.

Next, we measured MCP-1 and found significantly increased plasma levels in AF patients compared to healthy controls (268 pg/ml vs. 227 pg/ml; p = 0.03; Fig 2-A). However, MCP-1 baseline levels did not differ between patients with or without AF recurrence three months after PVI (281 pg/ml vs. 268 pg/ml; p = 0.86; Fig 2-B). In both groups MCP-1 plasma levels increased significantly after ablation independent form AF recurrence (patients without recurrence: 268 pg/ml vs. 349 pg/ml; Δ 81 pg/ml; p<0.01; patients with recurrence: 281 pg/ml vs. 355 pg/ml; Δ74 pg/ml; p = 0.03; Fig 2-C).

### Chromogranin A (CGA) plasma levels

**Fig 3:**
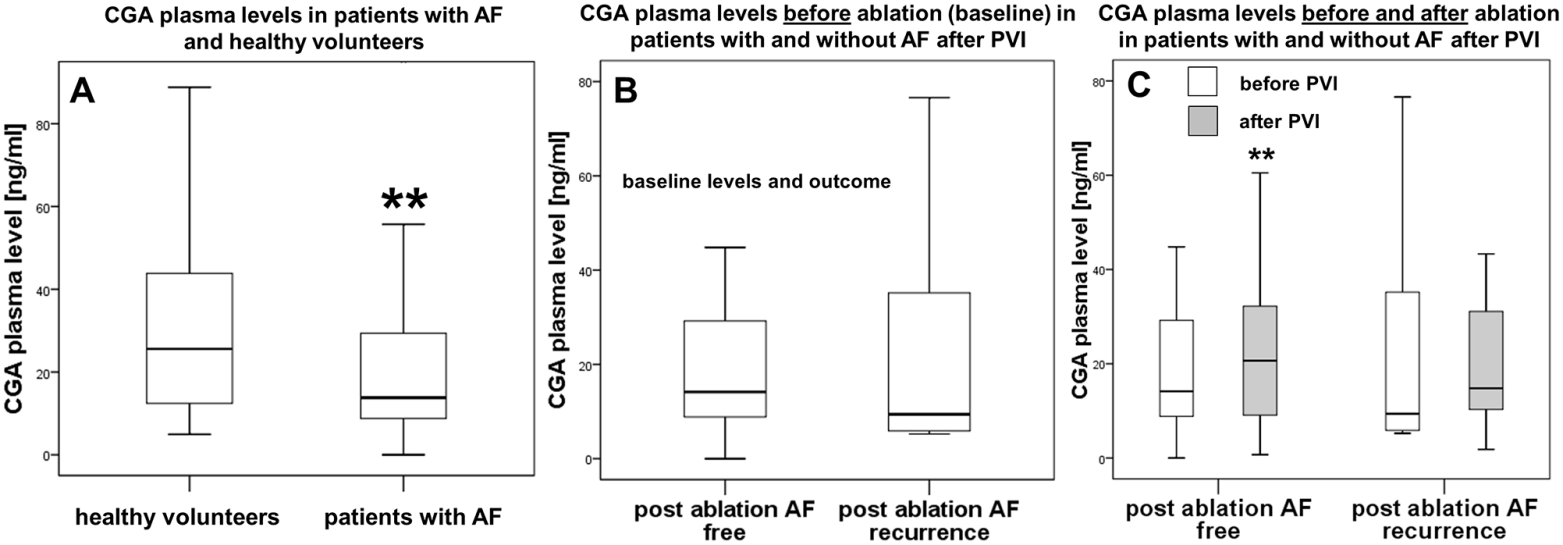
Plasma levels of CGA [ng/ml]. **A**, Healthy reference group without AF (left) vs. all AF study patients (right). **B**, Baseline CGA levels in patients without (left) and with AF recurrence (right) three months after ablation. **C**, CGA plasma levels before and after PVI in patients without (left) and patients with AF recurrence (right). Baseline levels presented in white bars, levels three months after ablation presented in grey bars. * p<0.05, ** p<0.01.

Patients with AF showed significantly lower plasma levels of CGA compared to our reference group (13.8 ng/ml vs. 25.6 ng/ml; p<0.01; Fig 3-A). We did not observe significant differences at baseline between patients with or without later AF recurrence (14.2 ng/ml vs. 9.4 ng/ml; p = 0.42; Fig 3-B). After three months CGA levels increased significantly only in patients without recurrence towards the level of healthy controls (14.2 ng/ml vs. 20.7 ng/ml; p = 0.008) but not in patients with recurrence (9.4 ng/ml vs. 14.8 ng/ml; p = 0.25; Fig 3-C).

## Discussion

In our current study we evaluated several blood markers of stress as potential biomarkers for atrial fibrillation and the association to AF burden/recurrence after an ablation therapy. The major findings of the present study were: (1) Patients with AF show marked differences with increased levels of ET-1 and MCP-1 and reduced levels of CGA compared to healthy controls; (2) in AF patients higher ET-1 levels were associated with AF recurrence three months after PVI; (3) freedom from AF after ablation was associated with a significant decrease of ET-1 and a significant increase of CGA three months after ablation compared to baseline.

### Endothelin-1

Our study shows that patients with AF have higher levels of Endothelin-1 compared to healthy controls without AF. This finding supports our hypothesis of a stress associated arrhythmia. However, previous reports about the relationship between ET-1 and AF are inconsistent. Some studies demonstrated elevated levels of ET-1 and proposed ET-1 being involved in remodelling processes and atrial dilatation [1,19]. In contrast, some clinical studies failed to show altered levels of ET-1 in patients with AF [20,21]. An examination of patients with AF receiving maze procedure could show that these patients show an increased content of ET-1 in left atrial appendage tissue correlating, among others, with atrial size and AF persistence [19]. Our findings suggest ET-1 as a predictive biomarker for the occurrence of AF after the ablation therapy. This is further supported by two reports by Nakazawa et al. and Wang et al. [1,22]. However, none of these studies showed a decrease of ET-1 after restoration of sinus rhythm as it is demonstrated by our study.

ET-1 has been shown to be associated with atrial structural [19] and electrical remodelling, e.g., by impairing calcium homeostasis [23]. Therefore, our findings could be in line with described effects of ET-1 in non-ischemic ventricular arrhythmia suggesting a direct pro-arrhythmic effect [24–26]. Nevertheless, in the context of our study it remains unclear if the altered ET-1 changes are actually a causal factor for AF or just a surrogate marker of a reduced stress level based on an arrhythmia free interval.

### MCP-1

Clinical trials have shown increased serum levels of MCP-1 in stress related cardiovascular disease [11]. In our study, patients with AF had higher levels of MCP-1 than healthy controls supporting our hypothesis that AF and stress might be correlating factors. Based on this hypothesis, treated patients without AF recurrence in the follow up period should present reduced MCP-1 levels. However, in our study we could demonstrate an increase after ablation in all treated patients independent from AF recurrence. A potential explanation for this is the also known proinflammatory effect of MCP-1 and its role in wound healing [27–29]. The goal of a PVI is actually to generate a localized scarring to achieve an electrical isolation of the pulmonary veins. Increased levels of MCP-1 could therefore be an indicator for an inflammatory and pro-fibrotic signalling as a response to the ablation procedure itself. Taken the fact that MCP-1 increase was, in contrast to ET-1, independent from AF burden after ablation, it could be hypothesised that MCP-1 change is likely to be more driven by the inflammatory response rather than by changes in stress levels. Furthermore, taken the observation that MCP-1 levels are higher in AF patients compared to healthy controls but failure to show an association with AF after ablation might also be interpreted as MCP-1 and AF being both related to stress as a common underlying factor but are not associated to each other in direct relationship.

### Chromogranin A

Circulating levels of CGA are influenced by cardiovascular diseases, which are also known risk factors of AF such as hypertension, heart failure, or acute coronary syndrome [4,30–32].

Nevertheless, no data is available on CGA in the context of AF. In our study we measured lower levels of CGA in patients with AF, a finding that does not directly support our hypothesis as AF being stress-related or vice versa. Given that the adrenergic system has huge impact on the development of AF [33], we expected higher levels of CGA as a surrogate for increased sympathoadrenergic tone. However, studies have also shown the importance of a balanced sympathetic/parasympathetic system since an increased vagal tone can also cause AF, that could at least partly explain the effects of ganglionated plexi ablation [34–39]. Low levels of CGA might be an indicator of a dysbalanced autonomic tone with an increased vagal tone and therefore an indicator or the causal fact of AF. There are data about patients treated with cryoballoon as well as radiofrequency ablation and additional ganglionic plexi modification (decreasing vagal tone) showing better rates of AF freedom [36,40–45]. This might be very speculative; however, this vagal modification resulting in a decreased tone of parasympathetic nervous system might thereby also translate in an increase of CGA (as a marker of sympathetic activity) after ablation. This would also explain the time course of CGA in our study. Patients without AF recurrence showed an increase of CGA (restoration of a balanced autonomic signalling) whereas patients with AF recurrence did not show a significant increase of CGA levels.

### Clinical implications and future perspective

Current success rates for PVI are reported with 60–70% after one year [18,46]. General use of more complex ablation strategies like additional ablation lines or CFAE mapping failed to improve success rates [47]. As a consequence individually tailored approaches of ablation seem necessary. Individual mechanisms like stress-related AF, vagal AF or obesity have to be considered to choose the optimal treatment. Our study findings support ET-1, MCP-1 and CGA as promising novel biomarkers for AF. Thus, they may help guiding the individual therapy and improving success rates in addition to other variables like MRI, genetic analysis, etc. [48–50]. The results of our study are hypothesis generating suggesting ET-1 actually being a marker for AF, MCP-1 might be stress- but not AF-related, while CGA being inversely related with AF speculating a role of vagal tone. Nevertheless, further studies dealing with the detailed role of these biomarkers are required to elucidate their value in a clinical setting.

### Limitations

A main limitation of our study is the short follow-up period of three months. In our study about 80% of our patients were still in sinus rhythm three months after ablation and a longer follow-up period might identify more patients with later AF recurrence. Our study results therefore only allow to draw conclusions regarding a short-term recurrence of AF or restoration of sinus rhythm but not a long-term ablation success.

To detect AF recurrence we used 24h Holter ECG recordings and patients’ reports on AF related symptoms as recommended by current guidelines. However, using those assessments we cannot detect AF recurrence in 100%, especially in case of asymptomatic episodes. The only alternative are implantable loop recorders that cannot be implanted routinely in all patients at the moment. Nevertheless, we think that this approach is acceptable for the purpose of our study, to evaluate stress biomarkers in the context of AF symptoms.

Furthermore, our study evaluated a heterogeneous population including patients with paroxysmal as well as persistent AF. Due to small sample sizes (especially in the persistent AF group) no separate analysis was possible. As a consequence we cannot rule out different biomarker patterns between these two entities of AF.

Further studies are necessary to put our findings in the context of a long-term follow-up and to explain the underlying pathophysiology. As an independent replication of our findings in a larger cohort is needed, our current study has to be called hypothesis generating potentially guiding strategies for future studies.

Nevertheless, to the best of our knowledge our study is the first to provide insights into the relationship and the absolute values of these stress markers to the presence or absence of AF before and after an ablation therapy as well as in comparison to a healthy control cohort.

### Conclusion

In this study we could provide evidence that established stress-related biomarkers ET-1, MCP-1, CGA were differentially regulated among patients with AF compared to healthy controls. Furthermore, the investigated biomarker levels before and three months after an ablation therapy revealed a direct association of ET-1 levels with respect to presence or absence of AF, and an inverse relationship of CGA levels regarding the presence of AF. These data provide new insight for the role of stress-related biomarkers in AF pathophysiology as well as potential biomarker for AF ablation therapy. However, larger, randomized, controlled clinical trials are necessary to finally prove clinical significance of those biomarkers and potentially allow to identify subgroups of patients at higher risk that might benefit from alternative ablation strategies like additional ganglionated plexi modification.

## Supporting information

S1 Table Patients characteristics are depicted. GFR, glomerular filtration rate; LA, left atrium.
(XLSX)

## Abbreviations

AF: atrial fibrillation
CAD: Coronary Artery Disease
CFAE: complex fractionated atrial electrograms
CGA: Chromogranin A
ET-1: Endothelin 1
GFR: glomerular filtration rate
MCP-1: monocyte chemotactic protein-1
NF-kB: nuclear factor kappa-light-chain-enhancer of activated B-cells
PLAX: parasternal long axis
PVI: pulmonary vein isolation
TNFα: tumor necrosis factor alpha
